# Salivary creatinine and urea analysis in patients with chronic kidney disease: a case control study

**DOI:** 10.1186/s12882-016-0222-x

**Published:** 2016-01-16

**Authors:** Taye Jemilat Lasisi, Yemi Raheem Raji, Babatunde Lawal Salako

**Affiliations:** Department of Physiology, College of Medicine, University of Ibadan/University College Hospital, Ibadan, Nigeria; 2Department of Oral Pathology, College of Medicine, University of Ibadan/University College Hospital, Ibadan, Nigeria; 3Department of Medicine, College of Medicine, University of Ibadan/University College Hospital, Ibadan, Nigeria

**Keywords:** Saliva, Plasma, Creatinine, Urea, Chronic kidney disease

## Abstract

**Background:**

Many metabolic changes develop in patients with chronic kidney disease which often necessitate frequent biochemical analysis of blood. Saliva analysis as an alternative to blood has many advantages. The aims of this study were to evaluate levels of salivary creatinine and urea in patients with chronic kidney disease in comparison to healthy individuals; to determine correlation between salivary creatinine/urea and blood creatinine/urea and to evaluate the diagnostic potential of saliva.

**Methods:**

A case control study, involving 50 patients with late stage chronic kidney disease and 49 healthy individuals as control. Blood and saliva samples were analyzed for urea and creatinine levels. Data are presented as median with interquartile range and compared using Independent Samples Mann Whitney U test. Correlation between plasma and salivary creatinine as well as urea was determined using Spearman’s correlation test. Receiver operating characteristics (ROC) analysis was done to determine the diagnostic ability of salivary creatinine and urea and cut-off values were established.

**Results:**

Median salivary creatinine levels were 2.60 mg/dl and 0.20 mg/dl while median salivary urea levels were 92.00 mg/dl and 20.50 mg/dl in patients with chronic kidney disease and controls respectively. Salivary levels of creatinine and urea were significantly elevated in chronic kidney disease patients (*p* < 0.001). In addition, there was positive correlation between blood and salivary creatinine as well as urea levels. Total areas under the curve for salivary creatinine and urea were 0.97 and 0.89 respectively. Cut-off values for salivary creatinine and urea were 0.55 mg/dl and 27.50 mg/dl respectively which gave sensitivity and specificity of 94 % and 85 % for creatinine; as well as 86 % and 93 % for urea.

**Conclusions:**

Findings of this study suggest that analysis of salivary creatinine and urea in patients with chronic kidney disease reflects their levels in blood. Hence, salivary creatinine and urea could be used as diagnostic biomarkers of chronic kidney disease.

## Background

Worldwide, increasing numbers of patients are affected by chronic kidney disease (CKD) [1, 2]. The progressive nature of CKD and the ensuing end stage renal disease (ESRD) is putting a substantial burden on global health-care resources [3]. Chronic kidney disease is associated with accumulation of metabolic waste products and multi organ involvements. These changes usually manifest as elevated blood urea and creatinine as well as hematologic, electrolyte, endocrine and skeletal disorders [4].

Several systemic diseases have been reported to produce marked and identifiable changes in salivary secretion [5–8]. Chronic kidney disease is one of the systemic diseases that can affect the contents of salivary secretions. More importantly, saliva can indicate creatinine and urea levels in patients with CKD which are the parameters usually assessed in blood samples. Analysis of salivary creatinine and urea in patients with CKD offers many advantages that have been attributed to the use of saliva as a diagnostic fluid.

Use of blood for diagnostic tests is an invasive process usually associated with nervousness and distress to the patients. Also, some form of blood loss is commonly related to procedures like hemodialysis and frequent blood sampling in chronic kidney disease patients [9]. In addition, the individuals involved in the management of CKD patients are at more risk of blood borne diseases. Hence, a non-invasive diagnostic test with minimal risk with ability to provide a dependable evaluation of disease condition would be of worth to both the health professionals and the patients.

Saliva as a biologic fluid secreted by the major and minor salivary glands plays the main role in oral health as well as systemic health. It has many advantages over serum because its collection is non invasive, simple, and requires minimal skill. Saliva sampling is appropriate for all age groups and can be repeated more frequently. It also offers a cost-effective method for the screening of large populations [10, 11].

Parameters in saliva can be affected by many factors including diet and genetics. Because of this, use of saliva as a diagnostic fluid is still subject to continuous research. This study was therefore designed to assess levels of salivary creatinine and urea; to determine the correlation between the levels in saliva and blood as well as to evaluate the diagnostic potential of saliva in assessing levels of creatinine and urea in patients with CKD.

## Methods

### Study design

This was a cross sectional survey of patients with CKD attending a tertiary hospital and healthy individuals as controls.

### Study population

The study received ethical clearance and approval from the institution Research Ethics Committee (University of Ibadan/University College Hospital Ethics Committee/13/0099). Patients with CKD were individuals diagnosed of the disease having estimated GFR of < 60 ml/min/1.73 m^2^ and stages 4 and 5 of National Kidney Foundation – Kidney Disease Outcome Quality Initiative (NKF-KDOQI) staging. The etiologies of CKD in the patients were hypertension, chronic glomerulonephritis, obstructive uropathy and diabetes mellitus. Most of the patients with CKD were undergoing dialysis treatment because of their late stage presentation. Also included in the study were the healthy controls who were volunteers and had no history of kidney disease, systemic or oral disease. Participants were provided information regarding risks and benefit of the study and verbal consent was taken. Participants had oral examination before saliva collection.

### Sample size

The minimum required sample size was calculated using the estimated means of salivary urea in a known test and control [12] with the standard normal values set at 0.05 and a power of 90 %. The calculated minimum sample size for each group was rounded up to 40 participants including 20 % attrition. Therefore, we included 99 participants (50 patients with CKD and 49 healthy controls) comprising 60 females and 39 males with a mean age of 39.45 years (range: 17 to 70 years). Patients with CKD were in stages 4 (12 %) and 5 (88 %). The demographic data of the patients with CKD and the healthy controls is shown in Table 1.

**Table 1:** Demographic of patients with CKD and healthy controls

	CKD patients	Healthy controls
N	50	49
Age (years)	39.82 ± 11.07,	39.07 ± 7.64,
	Range: 17 to 68	Range: 19 to 70
Male	20	19
Female	30	30

*N* Number of participants; Age is presented as mean ± SD

### Saliva and blood sampling

Saliva collection was undertaken throughout the day (between 9.00 h and 16.00 h) and participants had not had meal for at least 2 h before saliva collection. Whole saliva was collected by spitting method. Participants were asked to spit (after rinsing the mouth with distilled water) into calibrated universal plastic bottles until about 3 mls of saliva was collected. Saliva samples were stored at -20 °C until laboratory analysis. Samples were defrosted at room temperature and then centrifuged at 3000 rpm for 10 min before being used for the analysis in order to remove contaminants. Simultaneously, 5mls of blood samples were taken from the participants by venipuncture into lithium heparin bottles and the plasma was used for the analysis.

### Analysis of plasma and salivary creatinine and urea

Plasma and salivary creatinine levels were determined using modified Jaffe’s method [13] while urea levels were estimated using the method employed by Marsh et al. [14]. The methods involved colorimetric determination of creatinine levels using creatinine Assay Kit from RANDOX Reagents (USA) following manufacturer’s instructions. Levels of urea were also determined using Urea Assay Kit from RANDOX Reagents (USA). The absorbance was measured at 510 nm and 580 nm for creatinine and urea respectively using spectrophotometer-300 (ThermoScientific, USA). These methods have been standardized for saliva in previous studies [12, 15].

### Statistical analysis

The descriptive statistics of the participants’ demographic data are given as the mean and standard deviation while those of salivary creatinine and urea are presented as median and interquartile range. The outcome variables were median values of salivary creatinine and urea in patients with CKD and healthy controls. Values of salivary creatinine and urea were compared using Independent Samples Mann Whitney U Test (non-parametric test) because data was not normally distributed. Correlation between plasma and salivary urea as well as creatinine was assessed using Spearman’s correlation test. Linear regression equations were derived to estimate the plasma level of creatinine as well as urea from the salivary levels. Receiver Operating Characteristic (ROC) analysis was performed to evaluate the diagnostic potential of salivary creatinine and urea compared to blood and to correctly separate the participants into cases and controls i.e. to find whether salivary creatinine as well as urea levels can distinguish patients with CKD from healthy individuals (controls). The overall performance was assessed by the Total area under the curve and the cut-off values were determined based on the best trade-off between the sensitivity and specificity. All analysis was done using SPSS (version 22) and level of statistical significance was set at *p* < 0.05.

## Results

Plasma creatinine and urea levels are shown in Table 2. As expected the median plasma creatinine and urea were significantly higher in patients with CKD (*p* < 0.001).

**Table 2:** Plasma and salivary creatinine and urea levels in patients with CKD and healthy controls

		CKD patients	Healthy controls	*P* value
Plasma (mg/dl)	Creatinine	10.45 (5.85),	1.15 (0.42)	<0.001
	Urea	138.50 (126.00)	24.50 (13.25)	<0.001
Saliva (mg/dl)	Creatinine	2.60 (1.95)	0.20 (0.15)	<0.001
	Urea	92.00 (139.00)	20.50 (8.00)	<0.001

*Note*: Data are presented as median (interquartile range), *n* = 50 for patients with CKD, *n* = 49 for healthy controls

**Fig. 1:**
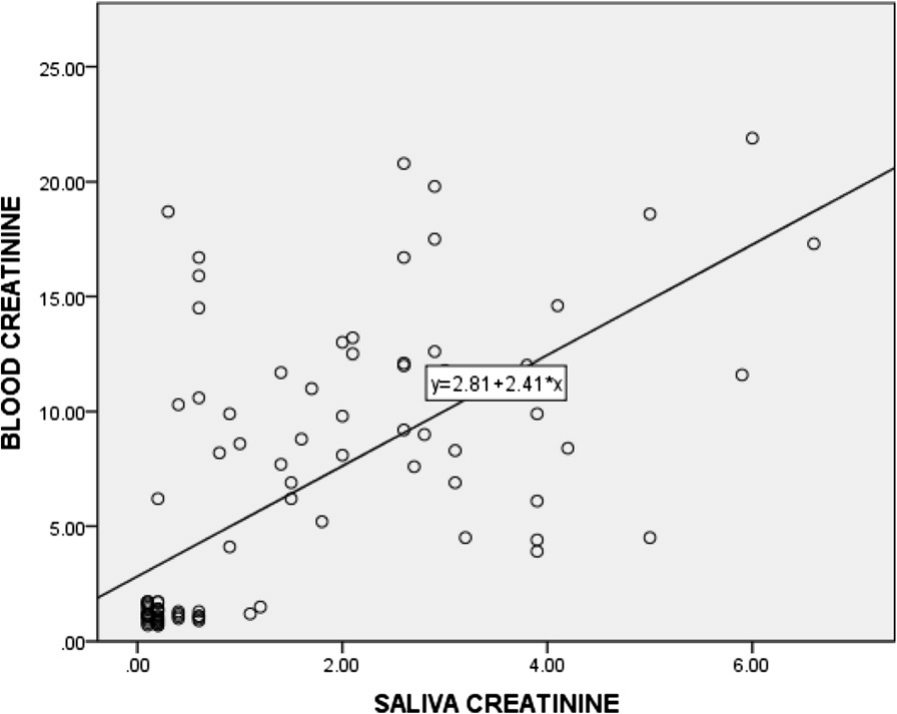
Correlation between salivary and blood creatinine levels in patients with CKD and healthy controls, *n* = 99

**Fig. 2:**
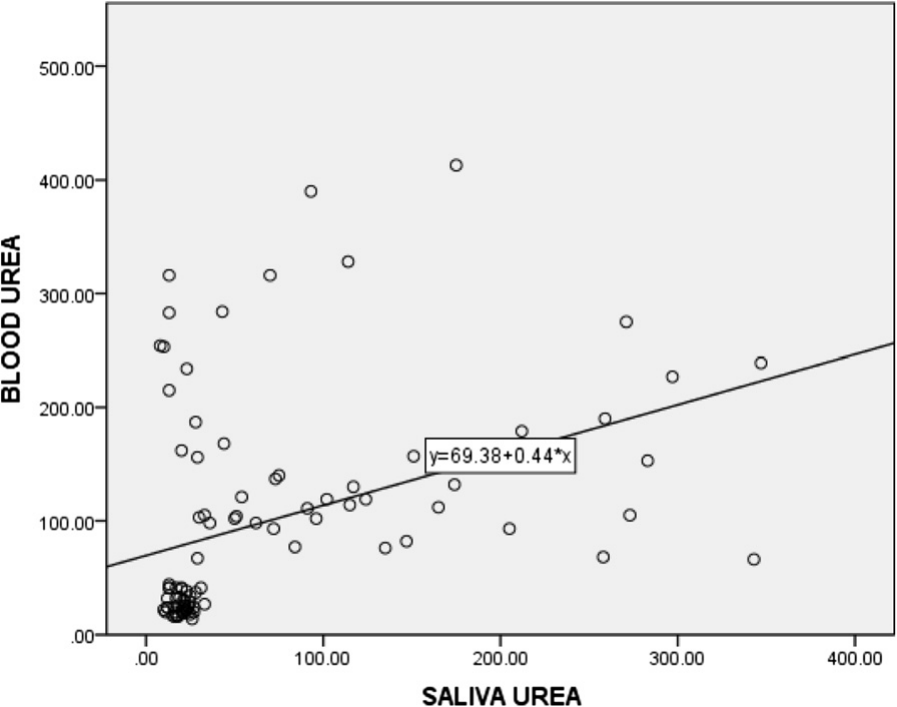
Correlation between salivary and blood urea levels in patients with CKD and healthy controls, *n* = 99

Correlation between plasma and salivary creatinine as well as urea showed a significant positive relationship (Table 3). Linear regression analysis performed to estimate plasma creatinine as well as urea levels in saliva showed the equations indicated in Figs. 1 and 2.

**Table 3:** Correlation between plasma and salivary creatinine and urea levels in patients with CKD and healthy controls

	Creatinine	Urea
Spearman’s rho	0.69	0.51
*P* value	0.00	0.00
N	99	99

*N* Number of participants

**Fig. 3:**
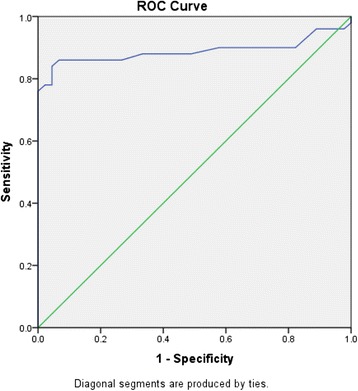
ROC curve for salivary urea levels. Total area under the curve for salivary urea is 0.89

To evaluate the diagnostic potential of salivary urea compared to plasma urea, i.e. to correctly separate the groups into cases and controls, ROC analysis (Fig. 3) was performed. Total area under the curve was 0.89 (Standard Error = 0.04, *p*-value < 0.001, 95 % confidence interval = 0.81–0.97). Sensitivity and specificity for different values of salivary urea were established and a cut-off was determined (Table 4).

**Table 4:** Coordinates of the ROC curve for salivary urea

Cut-off value	Sensitivity	1-specificity
0.00	0.00	0.00
26.50	0.86	0.11
**27.50**	**0.86**	**0.07**
28.50	0.84	0.04

Bolded data indicate the cut-off value for salivary urea based on the best trade off between sensitivity and specificity

**Fig. 4:**
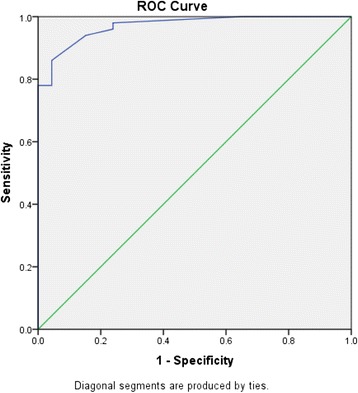
ROC curve for salivary creatinine levels. Total area under the curve for salivary creatinine is 0.97

Similarly, to evaluate the diagnostic potential of salivary creatinine compared to plasma creatinine, i.e. to correctly separate the groups into cases and controls, ROC analysis (Fig. 4) was performed. Total area under the curve was 0.97 (Standard Error = 0.01, *p*-value < 0.001, 95 % confidence interval = 0.944–0.998).

Sensitivity and specificity for different values of salivary creatinine were established and a cut-off value was determined (Table 5).

**Table 5:** Coordinates of the ROC curve for salivary creatinine

Cut-off value	Sensitivity	1-specificity
0.00	0.00	0.00
0.35	0.96	0.24
**0.55**	**0.94**	**0.15**
0.70	0.86	0.04

Bolded data indicate the cut-off value for salivary creatinine based on the best trade off between sensitivity and specificity

## Discussion

In this study, patients with CKD showed elevated levels of salivary creatinine and urea compared to the levels in healthy individuals. In addition, the salivary levels of creatinine and urea showed positive correlation with the levels in plasma. Our findings are consistent with previous reports [15–17] adding to the existing data to support the possibility of employing analysis of salivary creatinine and urea for the diagnosis and assessment of CKD.

The elevated levels of salivary creatinine and urea observed in patients with CKD are reflections of the blood levels as confirmed by the positive correlations. These elevated salivary levels of creatinine and urea could be responsible for the complaints of dry mouth [18], mouth odour or uremic breath [19] as well as tongue coating and other oral complications [20] of CKD. The uremic fetor, an ammoniacal odor from the mouth is a typical sign of uremic patients which is caused by the high concentration of urea in the saliva and its subsequent breakdown to ammonia [21]. Similarly, the positive correlation between serum and salivary creatinine observed in this study could be explained by the increased concentration of creatinine and urea in patients with CKD which creates a concentration gradient that facilitates increased diffusion of creatinine and urea from serum into saliva [22].

The positive correlation between blood and salivary urea as well as creatinine observed in this study agrees with previous reports [14, 23, 24]. In addition, a study by Tomas et al. [12] reported that concentrations of salivary urea were related to the severity of the kidney disease, and another study also showed that the concentration decreased with haemodialysis [25]. These suggest that analysis of salivary urea and creatinine could be an appropriate method for monitoring the efficacy of haemodialysis and progression of the CKD in addition to the use in the diagnosis of the condition.

Before salivary analysis of urea and creatinine can be adopted as a diagnostic method to replace the use of blood, the diagnostic value of the new salivary test must be compared with the available standard methods [16]. The accuracy of the new test depends on how well it separates the group being tested into those with the disease or without the disease [26]. Sensitivity and specificity are the basic methods to determine the accuracy of a diagnostic test. Hence ROC analysis is used to ascertain the diagnostic potential of a tool (in this case saliva) as an alternative to a standard method (in this case blood). The ROC analysis of salivary creatinine and urea in our study showed a good accuracy with good sensitivity and specificity. Our finding is in agreement with those reported by Ventakapathy et al. [16]. Thus, it implies that individuals with salivary creatinine as well as urea values above the cut-off values are more likely to suffer from CKD hence need further evaluation for appropriate management. In addition, the findings from the present study adds to the existing data that saliva can be used as alternative diagnostic fluid for estimating blood creatinine and urea in patients with CKD.

Some factors like age, gender, time of the day and meal affect salivary secretion. These factors must be considered in salivary analysis for it to be accepted as a diagnostic tool in assessing CKD patients. Some of these factors were also considered in the present study. In our study, saliva samples were taken throughout the day because studies by Peng et al. [15] and Cardoso et al. [27] have demonstrated that salivary urea has comparable concentration in the morning and afternoon. This implies that salivary urea does not significantly change during the day. They also suggested that salivary urea concentration is independent of saliva volume and detection of salivary urea can be done at any time during the day. In addition, it is particularly useful to monitor the condition of patients with CKD at all times, without the challenge of frequent blood sampling.

In our study, age matched healthy individuals were included as controls because studies [28, 29] have shown that age affects levels of blood as well as salivary urea. Renal function regresses gradually after a certain age like all other organs, so also the glomerular filtration rate leading to slight increase in blood and salivary urea as well as creatinine.

The general values for creatinine and urea in our study were higher than those reported by previous studies [15, 16, 24]. The variations can be attributed to the different populations, locations as well as ages of the participants. In addition, the higher levels of creatinine and urea observed in our study could be explained by the higher percentage of patients with CKD in stages 4 and 5.

One limitation of this study is the inclusion of patients in stages 4 and 5 of CKD (with higher proportion of stage 5 patients) only which was not intentional but could be explained by the late presentation of patients in our environment due to sociocultural and economic factors [30]. This also hindered the analysis of relationships between salivary levels of creatinine and urea with the stage of CKD. Another limitation of this study is the lack of different cut-off values depending on sex and age which could be explained by the sample size.

## Conclusions

Findings from our study demonstrated elevated levels of salivary creatinine and urea with positive correlation to the levels in blood in patients with late stage CKD. In addition, ROC analysis showed good sensitivity and specificity values of salivary creatinine and urea. This supports the possibility of using saliva in the diagnosis and monitoring of patients with CKD. However patients in the early and moderate stages of the disease must be included in other studies in order to consider salivary creatinine and urea measurement as a diagnostic tool for CKD.

## Abbreviations

CKD: Chronic kidney disease
ESRD: End stage renal disease
ROC: Receivers operating curve
SD: Standard deviation
